# COVID-19’s impact on faculty and staff at a School of Medicine in the US: what is the blueprint for the future?

**DOI:** 10.1186/s12913-021-06411-6

**Published:** 2021-04-28

**Authors:** Emily Gottenborg, Amy Yu, Roxana Naderi, Angela Keniston, Lauren McBeth, Katherine Morrison, David Schwartz, Marisha Burden

**Affiliations:** 1 Department of Medicine, Division of Hospital Medicine, University of Colorado School of Medicine, 12401 E. 17th Avenue, Aurora, CO 80045 USA; 2 Department of Medicine, University of Colorado School of Medicine, 12605 E. 16th Avenue, Aurora, CO 80045 USA; 3 Department of Medicine, Division of General Internal Medicine, University of Colorado, 12605 E. 16th Avenue, Aurora, CO 80045 USA; 4 Division of Pulmonary Sciences and Critical Care Medicine, University of Colorado School of Medicine, Aurora, USA

## Abstract

**Background:**

The Coronavirus Disease 2019 (COVID-19) caused unprecedented challenges within medical centers, revealing inequities embedded in the medical community and exposing fragile social support systems. While faculty and staff faced extraordinary demands in workplace duties, personal responsibilities also increased. The goal of this study was to understand the impact of the COVID-19 pandemic on personal and professional activities of faculty and staff in order to illuminate current challenges and explore solutions.

**Methods:**

Qualitative, semi-structured group interviews involved faculty and staff at four affiliate sites within the Department of Medicine at the University of Colorado, School of Medicine. Focus groups addressed the impact of COVID-19 on (1) Changes to roles and responsibilities at work and at home, (2) Resources utilized to manage these changes and, (3) Potential strategies for how the Department could assist faculty and staff. Thematic analysis was conducted using an inductive method at the semantic level to form themes and subthemes.

**Results:**

Qualitative analysis of focus group transcripts revealed themes of: (1) Challenges and disparities experienced during the pandemic, (2) Disproportionate impact on women personally and professionally, (3) Institutional factors that contributed to wellness and burnout, and (4) Solutions and strategies to support faculty and staff. Within each of these themes were multiple subthemes including increased professional and personal demands, concern for personal safety, a sense of internal guilt, financial uncertainty, missed professional opportunities, and a negative impact on mentoring. Solutions were offered and included an emphasis on addressing preexisting inequities, the importance of community, and workplace flexibility.

**Conclusions:**

The COVID-19 pandemic created burdens for already challenged faculty and staff in both their personal and professional lives. Swift action and advocacy by academic institutions is needed to support the lives and careers of our colleagues now and in the future.

## Background

Since the COVID-19 pandemic began it has become the disease with the highest mortality worldwide within a year [1–3] impacting nearly every geographic location worldwide and all sectors of society [3, 4]. In healthcare, COVID-19 has upended care delivery mechanisms, strained financial enterprises [4], and exposed the shortcomings of our emergency preparedness system [5]. In response, the medical workforce has had to adapt to extreme changes in the physical and structural norms of our work in order to provide necessary care to our communities [6–9]. Just as the pandemic exacerbated pre-existing racial, ethnic, and socioeconomic health disparities [10, 11], it has also intensified existing tensions in work-life balance. While all workers are facing increased stressors at home and in the workplace [12–15], women [16–22] are thought to experience even more demands on their ability to balance their personal and professional responsibilities.

On March 26th, 2020, in an effort to help curb the spread of the COVID-19 virus, the state of Colorado issued a public health order to close restaurants, bars, and other nonessential personal services facilities that was then followed by a stay-at-home order on April 9th, 2020, effectively closing schools and some day care centers [23]. The University of Colorado campus implemented work-from-home policies for non-essential personnel and research teams, restricting access to the medical campus to primarily clinical faculty, and requiring most staff to work from home. Some faculty members were redeployed from their research labs to serve as front-line providers in the hospital, and many outpatient providers transitioned entirely to virtual patient visits. Schools stayed closed through the remainder of the academic year with variability in reopening in the Fall of 2020. These adaptations that were commonly implemented across the country significantly strained faculty and staff: professionally, through new and ever-changing work conditions complicated by personal safety considerations, and personally, through increasing caregiving duties.

Previous literature has highlighted the negative interplay between work, gender, and caregiving [19, 24, 25]. Women have comprised the majority of the essential workforce during the pandemic in America with women representing 77% of essential workers in healthcare [26]. Women have also been shown to have a disproportionate share of domestic caregiving duties [27–31], which has been shown to impact academic productivity [32] and may impact career advancement. COVID-19 has added additional obstacles to promotion and leadership positions by introducing major changes and challenges to pre-pandemic social support structures. With the vast majority of primary and secondary schools converted to online platforms, caregivers with children found themselves adding schooling to the mix of work and home duties they needed to balance. This was compounded by the loss of previous family support structures (such as grandparents that help with caregiving duties) due to their high-risk for contracting a potentially lethal disease [33]. These increases in personal responsibilities further stressed a fragile system where pre-pandemic, frontline healthcare providers reported high levels of burnout, with some of the highest numbers in general internal medicine and emergency medicine [34].

Over the past decade, we have recognized the advantages of a diverse workforce and have striven to create one [35–41]. During the pandemic, increased demands at home combined with expanding clinical demands made academic productivity and advancement less attainable for these already disadvantaged groups.

As the COVID-19 pandemic upended social support systems, educational systems, and workplaces, swift action was necessary to understand how this new threat was impacting faculty and staff, and how to swiftly respond with support mechanisms and infrastructure to buffer the impact on our workforce. Utilizing qualitative methods, leaders across our department sought to characterize the personal and professional experience of faculty and staff during the COVID-19 pandemic and formulate solutions to address the issues faced.

## Methods

### Study design

Between July 28, 2020 and August 13, 2020, we conducted semi-structured focus groups via Zoom© with the Department of Medicine (DOM) faculty and staff at the University of Colorado (comprised of four separate sites: Denver Health, The Veterans Affairs Hospital, the Anschutz Medical Campus, and National Jewish Hospital). The “Consolidated Criteria for Reporting Qualitative Research (COREQ)” was used to guide the structure and reporting of the qualitative study results [42]. This project was approved by the Colorado Multiple Institutional Review Board and was deemed non-human subject research.

#### Setting and participants

Participants were recruited from all DOM faculty and staff (excluding the research team) across four sites as listed above at the University of Colorado. Multiple email invitations from both the DOM Chair and the research team were sent through a department-wide list serve and verbal invitations were also utilized at Department of Medicine town hall meetings. A convenience sample strategy was implemented due to the urgent need to identify areas of need and the constantly changing work environment that made participant availability unpredictable. Both faculty and staff were recruited due to the interdependent nature of their work and to provide a robust understanding of the experience and needs in the DOM community. Interested faculty and staff were scheduled for focus groups based on their scheduling preferences with the goal of having a minimum of 3 participants per focus group to ensure richness of conversation. Focus groups were conducted with concurrent analysis until we reached thematic saturation or no new themes were identified for at least 3 subsequent focus groups. Written consent to participate was sent in advance and verbal consent was obtained prior to the start of each focus group. Participants were informed at the start of each session that discussion moderators were also DOM faculty and/or staff and their shared interest in improving the academic working environment. In order to ensure anonymity, limited demographic data was collected.

#### Interview guide

A semi-structured interview-guide was used for each focus group and was designed to last for approximately 60 min. Questions explored the following domains: 1) Changes to roles and responsibilities at work and at home, 2) Resources utilized to manage these changes and, 3) Suggestions for how the department or divisions could help (Appendix). Questions were derived through a literature review as well as through summaries of free text from a preliminary survey that had been conducted in the Department of Medicine (survey was sent to 1209 faculty and 146 staff members with an 11% response rate). The interview guide questions were provided to a select group of faculty and staff to review prior to administration.

### Data collection

Study materials collected include audio recordings and transcripts, all of which were de-identified and stored in encrypted files on secure servers available through the University of Colorado HIPAA-compliant electronic shared folders.

### Analysis

Focus groups were audio-recorded and transcribed verbatim. Four female faculty physician team members moderated the focus groups (AY, EG, MB, and RN), and two female staff team members (AK and LM) took field notes. All members of the research team have experience conducting qualitative research; AK has a Masters in Science in Public Health and AY is completing a Masters in Clinical Science. Applying an inductive coding approach, team members (AK, LM and MB) developed a preliminary codebook after reviewing the transcripts and field notes with additional codes added as analysis was conducted. All team members reviewed field notes and coded focus group transcripts and consensus was established by identifying and resolving differences (AK, AY, EG, LM, MB, and RN). The thematic analysis was conducted using an inductive method at the semantic level, allowing themes to emerge from the focus groups [43]. Member checking [44], which is a technique for exploring the credibility of results, was conducted. All focus group members received a summary of the synthesized analyzed findings and findings were also circulated to leadership across the University for additional feedback.

## Results

Twenty-eight faculty and staff participated in the nine focus groups that were held from July 28, 2020 to August 13, 2020. Demographics are provided in Table 1. Themes and subthemes are discussed below.

**Table 1:** Demographics of faculty and staff within the department of medicine who participated in the focus groups

	***N*** = 28
**Gender, N (%)**	
Man (He, him)	4 (14.3)
Woman (She, her)	24 (85.7)
**Institution, N (%)**	
Anschutz Medical Campus	24 (85.7)
Denver Health	2 (7.1)
Veterans Affairs	1 (3.6)
National Jewish	1 (3.6)
**Employee type, N (%)**	
Faculty	24 (86.7)
Staff	4 (14.3)
**Academic appointment - faculty only, N (%)**	
Assistant Professor	9 (37.5)
Associate Professor	9 (37.5)
Instructor	3 (12.5)
Professor	2 (8.3)
Unknown	1 (4.2)
**Degree(s) (select all that apply), N (%)**^a^	
MD	16 (57.1)
DO	0 (0)
PhD	6 (21.4)
NP	0 (0)
PA	1 (3.6)
MPH/MS/MBA	6 (21.4)
BA	0 (0)
BS	0 (0)
Other	2 (7.1)
Unknown	1 (3.6)

^a^ Four individuals held multiple degrees

### Key themes

The following key themes emerged from the data: (1) Challenges and disparities experienced by faculty and staff during the pandemic, (2) Disproportionate impact on women personally and professionally, (3) Institutional factors that contributed to wellness and burnout, and (4) Solutions and strategies to support faculty and staff. Within each of these themes were multiple subthemes including increased professional and personal demands, concern for personal safety, a sense of internal guilt, financial uncertainty, missed professional opportunities, and a negative impact on mentoring. We will report each key theme and associated sub-themes.

#### Challenges and disparities experienced by faculty and staff during the pandemic

##### Increased professional demands

During the first shutdown, clinical and operational work increased and the demands on time for faculty and staff were strained. Staff and faculty were “burning the candle at both ends” to ensure personal and professional duties were met. Even when faculty/staff were ill, sometimes they would still be in service to their institutions.

*“I think we've all worked really, really hard. And we've met the deadlines by burning the candle at both ends. And now, we're going to deal with that.”* (Female, Interviewee 1, Focus Group 2)

Faculty and staff described that the nature of their work changed with an uncertain path towards advancement. They reported taking on additional clinical and administrative responsibilities, learning new technologies, forgoing academic productivity to secure their division’s financial solvency, and navigating how to do their work in unfamiliar environments.

*“In the early days, I was really heavily involved in our institutional preparation and response, and really worked I think in those first two weeks, 17 days of 12-hour days in a row. I didn't come home till nine o'clock at night, most nights, just trying to help our institution prepare. And then, immediately on the heels of that really long stretch, I got sick. And I was sick with COVID for about two weeks and was home up in my attic where I am now. I'm isolated away from my two kids and my husband, who's also a physician and whose work did not get lighter during COVID. And so, I think what kind of what happened in those early days, like all my other stuff got put on hold. And even when I was home sick, I was working probably 40 hours a week still just trying to help my institution be prepared.”* (Female, Interviewee 2, Focus Group 3)

*“There is such a push for clinical productivity because of finances, you know there’s a chance that our research is going to get put to the wayside...*” (Female, Interviewee 2, Focus Group 6)

##### Personal safety concerns

Frontline providers expressed initial concerns about the safety of others and their own families.

*“Her daycare actually stayed open during the entirety of the pandemic and I realize that that’s like an incredibly privileged place to come from, but we had opted to not send our daughter until mid-July– but paying a lot of money to not send my daughter with concerns of one, being the family that brings COVID into a space where there’s a lot of other children and families and then secondarily, concerned on our part about being a really concerning exposure.”* (Female, Interviewee 1, Focus Group 8)

*“I think I wore a bit of a scarlet letter when people knew that like I work at X Hospital, that that was like the hot, you know the biggest space for COVID patients that I think the perception was that I represented an increased risk to everyone which probably was true.”* (Female, Interviewee 2, Focus Group 8)

##### Stress and anxiety with shifting job responsibilities

The uncertainty about the duration of evolving changes created anxiety around defining the limits of one’s service to their institutions and reluctance to take on new opportunities.

*“I think in terms of the work responsibility, it has been hard, being a Hospitalist, COVID completely changed everything I knew and created an enormous amount of anxiety, particularly in March and April. We just didn’t know what we were doing and if it was going to keep us safe and so I think during that time of this year, there was this enormous stress and anxiety around just continuing to go into work and having – you know just caring for essentially, only COVID patients and having this huge loss of like what my job used to be.”* (Female, Interviewee 1, Focus Group 8)

*“I worried professionally that there are opportunities happening, that some people who are feeling overwhelmed and feeling the more burden of caregiving we are going to pass by.”* (Female, Interviewee 4, Focus Group 2)

##### Suffering from internal conflict

Finally, faculty and staff reported conflicted emotions around the anxiety, stress and challenges that they were facing, while acknowledging that others in the community were suffering to a much greater extent. They felt privileged to have a job, and were apologetic and guilt-ridden regarding sharing their experience.

*“I’m a big fan of this concept of dialectical thinking that you can at the same time be feeling the emotions of anger and frustration and sort of injustice about sort of sometimes how we feel we’re being treated and then on the other hand, recognize how fortunate I am and how you know positives that are going along at the same time, and that it – you know from a resiliency standpoint, it’s helpful to be able to acknowledge both levels of emotion and realize that you don’t have to live in one of those buckets*.” (Female, Interviewee 2, Focus Group 9)

##### Impact on mentoring

Mentors often expressed feeling overwhelmed by their own work and felt the impact of that for their mentees. Mentors felt that mentees also needed more support during the pandemic, but mentors often lacked the bandwidth.

*“Yesterday, I had a mentee send me a manuscript that she wanted me to review, and it was a mess. And then, I was like, “Well, is it a mess because I haven’t been helping you? But I didn’t know what you were doing?” Like, “I didn’t know what this was.” … I felt terrible because I’m supposed to be helping you, and I don’t have the energy”* (Female, Interviewee 2, Focus Group 3)

#### Women seem to be disproportionately impacted by increased caregiving duties

##### Caregivers with children and elder care responsibilities are struggling

Faculty and staff reported that their domestic roles and responsibilities dramatically increased during the COVID-19 pandemic, which was reported by female participants in particular. They specifically pointed to the demands of in-home education (keeping up with academic content and helping children adapt to online learning), full-day childcare, managing day-to-day needs for aging family members, and household work. Some healthcare providers reported difficulty in finding help because they were perceived to be at high risk for COVID-19 transmission.

*“It was really, really hard to fit all of that in. We are not trained teachers. I don’t know how to teach elementary school, and you know I’m trying to do my regular work during the day because that’s what everybody else is expecting -- to hear back from me and have action happen but then that means that I have to basically put her school on the back burner until later in the afternoon. So, I would start work at like five or six and then I would try and knock off at least by two or three so that I can then help her with school but by that time I’m fried. Lots of tears, you know.”* (Female, Interviewee 1, Focus Group 4)

While most focus group participants described increasing stress with these new responsibilities, some faculty and staff found them as opportunities to reconnect with their children or elderly family.

*“I think my kids are really enjoying being home with mom and dad as much as they are. When we first started going back to the hospital after that initial complete lockdown phase, my kids were flabbergasted that I was leaving the house. “No, mommy. Don’t go.” I definitely think the bond is much stronger now”*. (Female, Interviewee 2, Focus Group 5)

##### Missed opportunities

Staff and faculty reported diminishing reserves and abilities to take on new duties; most notably in women who expressed taking on more caregiving duties. There was a reluctance to look for new opportunities given the many uncertainties, risking a deepening gender disparity in faculty advancement due to caregiving duties.

*“I'm trying to do too much. It's all taking longer. I think from a professional point of view, I’m really rethinking opportunities that maybe a year ago I would have jumped at. And I'm sort of in a, I just can’t rock the boat situation. I’m barely keeping my head above water. I can’t take on a newer responsibility. Like let’s just keep things the way they are. Keep my schedule the way it is. And there’s a lot of neat educational opportunities coming up. I worried professionally that there are opportunities happening, um, that some people who are feeling overwhelmed and feeling the more burden of caregiving are going to - or are going to pass by.”* (Female, Interviewee 4, Focus Group 2)

*“I was doing my MBA program and decided to take probably this next year off because I feel that any extra time, I need to make sure that my kids are keeping up with their academics, and that’s sort of where the priority for everything is gonna go this year … you feel those pulls. Even if you are performing and sort of outwardly functioning, internally you really do feel it.”* (Female, Interviewee 2, Focus Group 7)

##### Flexibility in work (i.e. timelines and advancement)

Faculty frequently brought up stress surrounding promotion timelines, with many expressing fear of missed opportunities for advancement due to competing workplace and personal responsibilities. Faculty involved in research or scholarship reported stress about meeting grant timelines and fear about job security given delays in productivity. Lack of perceived flexibility regarding these timelines and transparency about available support mechanisms added to faculty stress.

*“I will say that’s probably my biggest fear from a career perspective ... is that because I have kids and because people recognize that you know there has to be more flexibility and that I may not be available at the same time, that I will not be given opportunities down the road that I otherwise would have received or been able to – you know to apply for. I will be viewed in a different light because of how this has changed my ability to be present. I don’t think it’s that the work product itself is significantly less, but it’s just my availability is not the same– that’s a huge fear of mine.* (Female, Interviewee 1, Focus Group 7)

*“Fundamentally I know I am underpaid compared to my colleagues and that pisses me off. And so, I don’t want to delay (promotion) … I’d like to have the option to delay if I get to it and realize, I’m not where I need to be. But I don’t want to defer getting an increased salary for a year just because I might need that.* (Female, Interviewee 3, Focus Group 1)

*“I think that (one) year’s worth of grace period is actually quite helpful. My promotion clock timeline is right there so it was helpful. I don’t know if I’ll use it or not. I think honestly, part of it is just the feeling of we’re all overachievers. We’re all in academic medicine for a reason, and feeling like we’re not able to be productive in that way is just uh – I think it’s a personal, self-induced stress as well, so I don’t know that the department necessarily is going to be able to smooth that any more than they already have.”* (Female, Interviewee 2, Focus Group 5)

#### Institutional factors were identified that contributed to faculty and staff stress and well-being

##### The untenable nature of a return to “Normal”

Frontline faculty and staff expressed concern over the ability to maintain their professional duties while concurrently caregiving at home, especially as there has been a shift toward returning to a new normal of day-to-day operations. Interviewees described feeling mounting pressure to return to their previous level of productivity, given that their mentors or leaders assume they have more time since on-campus activities have lightened. This messaging was upsetting because of the lack of recognition of increased domestic duties. They also described that the blurring of personal and professional boundaries has created longer days, exacerbating unrealistic expectations of academic productivity.

*“I think a huge thing is there’s not a separation between work and home at all anymore. It just kind of all blends together. And I feel like the evening hits and it's only when the kids complain how hungry they are that I think, "Okay, I got to stop and make dinner." There's not that like, "I'm home from work now. Let's have a little family time before I go back to work." It's just, you keep working until - until you don't.”* (Female, Interviewee 4, Focus Group 3)

*“If the system was challenging and this close to breaking to begin with, then I think the idea of going back to the way things were is a futile and misguided effort*.” (Male, Interviewee 4, Focus Group 1)

*“It just seems to me now like it's a gas, everything is a gas, and it's just expanding to fill the space. And so, there are no more boundaries of when I'm doing one thing or the other thing. It's like I'm doing laundry while I'm writing while I'm scheduling while I'm in between talks I'm giving, and all of it is just filling all the space.”* (Female, Interviewee 2, Focus Group 3)

##### Financial uncertainty

Faculty and staff both discussed the stresses brought about by competing interests to maintain financial solvency both at an institutional level as well as at a personal one. Institutionally, a focus on generating increased clinical revenue has shifted support from other academic enterprises, and personally, changing educational platforms (online versus in-person versus hybrid) and child care options make anticipated costs difficult.

*“Then it leaves me with the option of either going down over all in terms of my salary, or to keep my salary, it means that I would increase my clinical role, which I’m not opposed to doing but given the uncertainty. I think the probable last-minute changes that will have to happen, it doesn’t seem like that’s necessarily the most responsible or respectful option but on top of all the financial burden of trying to figure out the childcare and the pieces of that, I’ve also had a cut in my salary. Again, and I don’t want to sound selfish or ungrateful in any way because I know that a lot of people have it much worse. Fortunately, I think I will have a job through all of this … ”* (Female, Interviewee 1, Focus Group 7)

*“I do think that there is a stressor about job stability, and there’s a big push and pull with you know we have got to work. We have got to keep working or working more in order to stay afloat.”* (Female, Interviewee 1, Focus Group 5)

Some benefits were also noted in that novel reimbursement models have emerged.

*“One of my bright spots is X funding agency always told us in primary care, we won't pay you for telephone visits, right? And then one day in March, they said, “We'll pay you for telephone visits”. And I was like, “Darn it, if we can make something as hard to scale as you know the – X funding agency change” -- then we need to make the most of them. We need to do things.”* (Male, Interviewee 1, Focus Group 1)

##### Virtual health challenges

Outpatient faculty and staff described initial challenges with virtual healthcare delivery platforms. There were also concerns with trying to get patients back to in-person visits too quickly.

*“There was a lot of back and forth in June about “Hey, when are you coming back? Hey, we want to have you see patients in person” and I pushed back pretty hard and it was like, “Guys, who wants to be seen in-person?” We opened up a half day of my clinic just to experiment with it and nobody signed up for any in-person visits and we were doing reasonably well by June in terms of telehealth and worked out a lot of the kinks.”* (Male, Interviewee 4, Focus Group 4)

#### Local solutions and strategies to support faculty and staff

##### An opportunity to address pre-existing inequities

Faculty and staff alike recognized opportunities that arose during the pandemic. Processes and procedures that would have taken time to develop were rapidly instated, eliminating some of the bureaucracies of within our healthcare systems. Participants were hopeful that our systems are not put back together as they were before.

*“This radical disruption or radical innovation, we should really seize on this, this is an opportunity and we can right some wrongs. And I think if we open ourselves up to that, I think that we start to tackle our vast inequity problems in terms of gender and our huge glaring diversity problems. And we've made great strides in the 14 years I've been faculty, but those strides are so minimal in comparison to the journey that we have to take on those lines.”* (Male, Interviewee 1, Focus Group 1)

Other areas of focus for solutions included offering resources for parents/guardians for school-age children and elder care, supporting innovation in defining the new normal, allowing flexible timelines for promotion and grants, supporting virtual healthcare delivery services, and offering reassurances around financial investment in faculty and staff (Table 2).

**Table 2:** Proposed solutions directed towards areas of most concern, provided by focus group participants

Area of Concern	Recommendation	Resources
Parents/guardians with school age/younger children	Emergency child care services (back up, crisis care) Daycare options Educational support	Partnership with local companies and schools; stipend support for those in need; administrative support for coordination of learning groups; ability for employees to pool sick/vacation days for those in need; matching programs (i.e. database to connect faculty and staff with similar needs)
Elder care support	Support groups such as psychologist/peer groups/expert groups (geriatricians/palliative care) to help with planning and experiences	Time for support groups; matching programs
Returning back to “normal” to quickly	Develop return to work plans that are innovative and harness the positives from current experience	Divisions to collaboratively work with their teams to develop revised work policies that promote workplace flexibility and sustainability
Flexibility (i.e. timelines for funding agencies, promotions, and work schedules)	Work with funding agencies to create flexible timelines/understanding of options with grants; offer deferrals/support/time to prepare dossiers for promotions when needed	Designate institutional lead to work with funding agencies on innovative and supportive timelines; develop expert panel; offer additional support for dossier preparation; flexibility with work schedules
FMLA/PTO for COVID illness	Message faculty and staff about already existing benefits	N/A
Financials and other uncertainties	Clear communications; continued transparency	N/A
Resiliency/coping	Support programs	Support programs; advertisements of offerings; acknowledging the struggles and potential solutions through focus groups

##### Enhanced sense of community

While faculty and staff were eager to offer focused solutions for various challenges, most also described the need to leverage the sense of community that arose from the pandemic.

*“One of the positives that has certainly come out of this from my perspective is the community, the sense of community. Early on one of our colleagues reached out and said, ‘Anybody who needs a break,’ and it was to the moms. “Anybody who needs to give up a shift to be able to stay home with your kids let me know. I’m happy to help.” And so it was just really thoughtful and created a really nice sense of community that we’re all in this together. Everybody’s struggling with the same things, so I think from that perspective, I’ve really seen positive changes.”* (Female, Interviewee 1, Focus Group 7)

## Discussion

The important findings of this study are: (1) the pandemic has created new and exacerbated existing challenges and disparities experienced by faculty and staff at an academic medical center, (2) women seem to be disproportionately affected by increases in caregiving duties that may be impact their promotion and advancement trajectories, (3) overarching institutional factors have contributed to faculty and staff stress and well-being; and (4) there are multiple solutions that our institution can implement to help support these critical members of our workforce.

All faculty and staff interviewed felt significant impacts in their professional and personal lives. Early literature on the impact of the COVID-19 pandemic suggests that women and junior faculty may be at higher risk for negative impacts on their careers, burnout, and career longevity. The majority of our participants were women and more junior in their career path. Literature in the lay press has taken note that women have been adversely impacted by the pandemic; women have been the predominant frontline response to the pandemic, yet have faced disproportionate increases in caregiving duties [18, 20, 21], and this is contributing to increased burnout and stress [21]. Prior to the pandemic, women already faced disparities in promotion, leadership roles, recognition, authorship, pay, and speaking opportunities [40, 41, 45–54]. Early reports are already showing that women have seen a reduced rate of publication submissions and research productivity [55–57] since the COVID-19 pandemic started.

Previous work has shown that male faculty are four times more likely than women faculty to have a partner that is engaged in full time domestic activities and has highlighted the disproportionate share of caregiving duties on women [58]. The COVID-19 pandemic has further challenged those with caregiving duties, especially those with young children, who have had to subsequently move much of their dependent’s educational activities to online learning. Furthermore, pre-pandemic, other family members such as grandparents often contributed to childcare, but our work highlights that these support systems were also fractured with COVID-19 since elderly caretakers may no longer be able to care for children given they were in higher risk categories for adverse impacts from COVID-19.

Career advancement and promotion were common topics of concern and similar to above, there was concern that women and faculty that have caregiving duties may be less inclined to take opportunities. Some potential solutions that emerged from the focus groups include continuation of alternate and flexible work schedules, development of flexible promotion timelines, investment in family support mechanisms, creation of social support networks to leverage a sense of community, and utilization of the momentum for change to address pay gaps due to gender. Our findings add to the current literature of how to best support faculty during the COVID-19 pandemic [59], which has been primarily recommendations and expert opinions, by providing real-world experiences from front-line faculty and staff. Furthermore, in our preliminary survey data [data not published], a disproportionate number of women were considering delaying promotion due to increased professional and personal demands though it is unclear if those trends will definitely result in actual delays; current promotion and tenure policies with an option to delay due to the challenges are a common consideration [60], however, while well-intentioned, could also have adverse impacts, particularly if salary is linked to years in rank. Previous work has also highlighted that such policies substantially reduced female tenure rates while substantially increasing male tenure rates [61]. Thus ensuring that these well-intentioned policies lead to eliminating inequities as opposed to worsening them will be key.

There were also some positive changes related to the pandemic noted by faculty and staff, such as more flexible work opportunities and schedules and an increased ability to attend various meetings due to remote capabilities. Previous work has highlighted how women in particular can be disadvantaged in the academic world as it increasingly requires availability and mobility for networking and conferences, which has diminished over the past year due to the pandemic [24].

The unifying urgency for support underlying the sentiments of our focus group participants was most resounding, regardless of the specific needs identified by respondents. Increasingly, institutions and organizations are starting to share their best practices as it pertains to occupational safety and at-risk faculty [59, 62].

Our work has several strengths. This is one of the first reports coming from a major academic medical center that aims to understand the perspective of faculty and staff from four separate institutions. Our research also has several limitations. Our focus groups were conducted fairly early during the pandemic and thus perspectives have likely shifted as organizations have started to address the various concerns raised. Even though all faculty and staff were invited to participate, there were a disproportionately higher number of women who participated in the focus groups, suspected in part because of women’s predominant roles in caregiving and possible lack of self-identification of men as caregivers, though our initial intent was to focus on both men and women. We did not ask about issues related race or ethnicity in our focus groups though some previous work has highlighted the intersectionality of gender and ethnicity and thus there may be additional inequities and issues specific to certain subgroups [19]. We did aim to ensure as diverse of opinions as possible by inviting all Department members to the focus groups; future opportunities include exploring perspectives of learners.

## Conclusions

The COVID-19 pandemic has brought to light the heavy burdens on faculty and staff, many of whom are frontline responders. Women seem to disproportionately face increased caregiving and household duties, and this data suggests the pandemic may impact their opportunity for promotion and the trajectory of their careers. There are numerous solutions that could be implemented to help mitigate the impact of COVID-19 on faculty and staff such as continuation of alternate and flexible work schedules, development of flexible promotion timelines, investment in family support mechanisms, creation of social support networks to leverage a sense of community, and utilization of the momentum for change to address pay gaps due to gender.

## Data Availability

The survey and qualitative data generated and analyzed for this study are not publicly available in order to preserve participant confidentially and meet regulatory requirements from the authorizing institutional review board. Data may be available from the corresponding author upon reasonable request.

## Appendix

### Caregiving in COVID-19 Department of Medicine – Focus Group Interview Guide

1. Tell me about how your day-to-day life, both home and work, has changed, both positive and negative, as a result of COVID-19?
  - Probe a: Do you have extra roles at work or at home?
  - Probe b: Is your productivity affected? Are there obstacles to getting things done either at home or at work?
  - Probe c: Is anything being delayed? Grants? Papers? Promotion?
  - Probe d: Are there any challenges to communication? Facilitators to communication?
2. Tell me about the resources or people you have relied on to help manage your responsibilities both at home and at work
  - Probe a: What worked best?
  - Probe b: What did not work well?
  - Probe c: What was missing?
3. What is the one thing that could be done at the department or division level to help during this time?
4. Is there anything I did not ask you about that you think is important for me to know?

## Abbreviations

COVID-19: Coronavirus 2019
HIPAA: Health Insurance Portability and Accountability act of 1996
